# Improving LISA Practice: An Ongoing Observational Quality Improvement Initiative Following Initiation of Less-Invasive Surfactant Administration in a Level IV NICU

**DOI:** 10.3390/children13040571

**Published:** 2026-04-20

**Authors:** Tynisha Koenigsaecker, Shreya Patel, Stephanie C. Martinez, Kevin Ives, Julie Bodie, Chad Weagraff, Monika Bhola, Rita M. Ryan

**Affiliations:** 1 Department of Pediatrics (Neonatology), Rainbow Babies and Children’s Hospital, Case Western Reserve University, Cleveland, OH 44106, USA; tynisha.koenigsaecker@uhhospitals.org (T.K.); shreya.patel@uhhospitals.org (S.P.); kevin.ives@uhhospitals.org (K.I.); julie.bodie@uhhospitals.org (J.B.); chad.weagraff@uhhospitals.org (C.W.); monika.bhola@uhhospitals.org (M.B.); 2 Department of Pediatrics (Neonatology), University of Tennessee Health Science Center, Memphis, TN 38163, USA; smarti71@uthsc.edu

**Keywords:** prematurity, surfactant, respiratory distress syndrome, video laryngoscopy, endotracheal intubation

## Abstract

**Background/Objectives**: Surfactant has been delivered via less-invasive surfactant administration (LISA) in our neonatal intensive care unit (NICU) since 2020. Data have been monitored and the literature regularly reviewed to improve our LISA practice. The purpose of this project is to share the clinical practice changes made to help other NICU providers fine-tune their LISA practice. **Methods**: The original LISA criteria included babies with GA 27–36 6/7 w, on > 21% O_2_, on continuous positive airway pressure (CPAP), pCO_2_ < 70 if a blood gas was obtained, and radiographic and/or clinical evidence of respiratory distress syndrome (RDS). Current criteria include GA 25–35 6/7 w and minimum CPAP + 6. This manuscript highlights the changes made since 2023. To monitor these changes, targeted data from the entire cohort were examined before and after each change. **Results**: LISA was attempted on 399 babies (average (SD) GA 31.7 (2.7), birth weight 1752 (590), with a procedural success rate of 97%. Overall, 18% required intubation within 7 days after LISA. The median (IQR) for FiO_2_ was 32 (28, 40) prior to LISA and 23 (21, 30) post-LISA and the hour of age of LISA was 4 (2.5, 9.9). LISA procedure success rate was increased by the use of video laryngoscopy as well as reinforcement of the use of sucrose sedation and swaddling; our first attempt success increased overall from 39% to 52%. After the introduction of a clinical RDS score (Downes), there was an expected and logical increase in the number of infants requiring intubation within 7 days of LISA indicating likely over-treatment prior to this change. After implementation of a clearly described plan for babies <28 w gestation there was a decrease in the hour of age of LISA from 3 (2.5, 4.5) to 2 (0.8, 3) h. **Conclusions**: It is critical to continually evaluate a new practice and identify strategic changes. We offer our changes to assist others starting or using LISA.

## 1. Introduction

The LISA procedure, prevalent and well-established outside of the USA, is still in its infancy here. It should be noted that we use the term LISA broadly to describe our method of surfactant administration which in our unit is technically the Minimally Invasive Surfactant Therapy (MIST) Hobart method [1]; other authors have done the same [2]. The LISA journey in our NICU began in June 2020 after gathering equipment, training team members, and preparing a protocol. As described previously [3], we use “LISA kits” that include a 16 Fr angiocatheter, a sterile marker, a sterile ruler, a blank sterile label for a “flag,” sterile gloves, and a procedure guide. The procedure guide includes our eligibility criteria and general procedural guidelines on one side, and on the reverse side, a space for the nurse in charge (required for every LISA procedure as an extra set of hands and to fill in potential nursing knowledge gaps) to document all pieces of the procedure as well as collect some small amount of data, such as FiO_2_ before and after LISA, adverse events, etc., in real time. Based on the prevalence of LISA outside of the USA [4] we knew there was evidence that LISA was being used elsewhere and was preferable to endotracheal tube (ETT) surfactant delivery [5]. We contacted several other large USA NICUs who were all “thinking about” starting LISA or were “developing” their protocol, but no one had an active LISA protocol to share, so we created and started using our own protocol and published our experience [3] to help other NICU teams who were starting LISA. That strategy generated positive feedback, so we are now sharing our experience with how our LISA program has evolved over time.

The LISA steering team comprises neonatologists, neonatology fellows, NICU respiratory therapists (RTs), a pharmacist, and a neonatal physician’s assistant. It has been critical to have the commitment of our RTs who add consistency from baby to baby and help reinforce adherence to our protocol. The team meets monthly to review data, discuss the literature regarding surfactant administration, plan, and problem-solve. All protocol changes are presented to the neonatology divisional quality improvement (QI) group and the bronchopulmonary dysplasia (BPD) QI group. Additionally, all changes are presented in writing with data and literature to support the change. Changes are made collaboratively with nursing and RT teams to optimize success.

Data have been collected continuously by the fellows on the LISA steering team and monitored at our monthly meetings, along with regular review of the relevant literature, to make improvements. Each change made was for a specific reason. This information is being shared for both those who are introducing LISA into their NICU and for those who are continuing to use LISA and considering new improvements.

## 2. Methods

**Figure 1:**
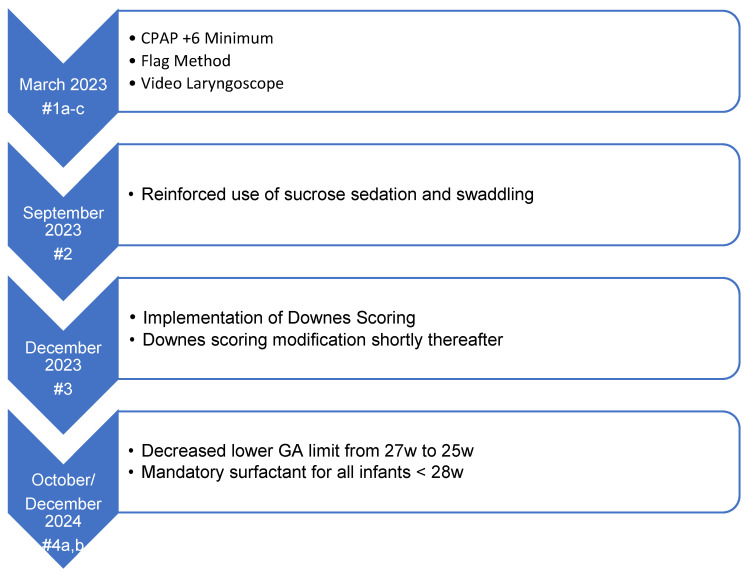
A timeline of strategic changes implemented for continuous improvement of our LISA practice.

This project is a descriptive, retrospective, observational quality improvement initiative involving sequential, non-randomized protocol modifications. The decision for each LISA protocol change (Figure 1) made was based on the literature and national discussion, equipment availability, and how quickly we thought we could realistically implement each change. The Institutional Review Board of University Hospitals (Rainbow Babies & Children’s Hospital, Case Western Reserve University) determined the study to be quality improvement and non-human subjects research.

Data were collected from the beginning of LISA implementation (June 2020 to October 2025). However, the data were collected as time permitted and not always immediately after a change, so while we did not refer to these changes as Plan-Do-Study-Act (PDSA) cycles, we did examine our data often at our monthly meetings. Each time we made a LISA protocol clinical practice change (Figure 1) we discussed the specific data measure outcome we wanted to examine that we thought would be most relevant to that change. All infants who had LISA attempted were included. Infants were “LISA-eligible” if they met LISA criteria; this was defined initially as gestational age GA 27–35 6/7 w, on >21% O_2_ CPAP. We collected data documenting typical adverse events during the LISA procedure itself. These were defined as: bradycardia (heart rate < 60 for ≥5 s), desaturation (SpO_2_ < 60%), cough, surfactant reflux, emesis, trauma, and need for emergency intubation during LISA.

Categorical data were analyzed by chi-square or Fisher’s exact test. Most continuous data were not normally distributed and were examined by the Wilcoxon rank sum test. A *p* value < 0.05 was considered significant. Each clinical practice LISA “change” was evaluated by a new comparison of the data for all babies before and after the change temporally so that each evaluation was composed of a unique comparison but included all cumulative groups of infants before and after the change.

Some changes occurred at the same time (e.g., #1a–c) or very close in time (e.g., #4a–b) and so were combined for analysis because there was no way to separate them, or the n was too small to make a realistic comparison.

### LISA Clinical Practice Changes—Rationale and Implementation

Change #1a—LISA Criteria Minimum CPAP increased to +6; March 2023
Rationale

Our initial practice of placing the babies on CPAP + 5 was changed to CPAP + 6 based on the RDS-NeXT recommendations [6] and the success of OPTISURF [7].

Implementation

Implementation included education with RTs, fellows, and faculty regarding standardization to +6 CPAP for LISA eligibility. The compliance with this was monitored on the LISA kit form.

Change #1b—Flag method to reinforce endotracheal tube distance; March 2023
Rationale

Despite using a marker to place a line at 2 cm from the tip of the angiocath [3], infrequently it appeared that the surfactant went predominantly to the right lung and so we implemented a method to improve the chance of bilateral surfactant delivery every time.

Implementation

**Figure 2:**
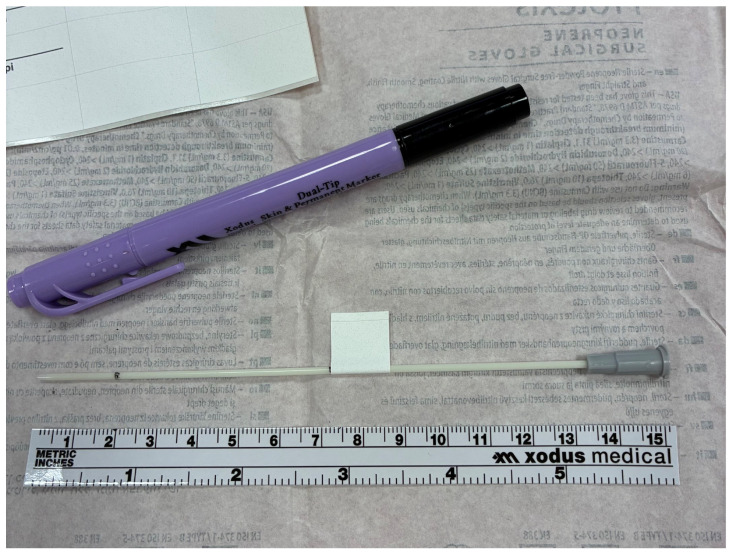
To prevent migration of the angiocatheter, a sterile label is placed as a flag at the estimated depth for intubation based on weight. The flag is not permitted to enter the mouth during LISA, thus avoiding unilateral surfactant delivery.

To improve consistent bilateral surfactant delivery, the use of a flag method (Figure 2) was implemented which allows for the establishment of proper catheter placement by measuring out a catheter insertion depth (identifying where intubation depth in centimeters occurs on the catheter, and marking the point off with a sterile label folded onto itself around the catheter). The flag serves as a marker to be held in place just outside the lip of the neonate during surfactant administration. This is not meant to replace the line marked on the distal end tube to be placed at the cords, but to supplement it while the surfactant is being instilled to remind the LISA provider to avoid migration of the catheter lower into the trachea by keeping the flag outside the lip.

Change #1c—Introduction of Video laryngoscopy; March 2023
Rationale

Placement of an angiocatheter for LISA requires rigorous visual confirmation [2] and the use of a video laryngoscope (VL) has been demonstrated to improve first attempt success intubation rates in neonates [8,9,10,11]; in our own unit, we found VL improved first attempt intubation success, from 45% to 57% [12].

Implementation

Initially, a single VL device (NeoView Video Intubation System, Austin, TX, USA) was procured. Simulation sessions were conducted for faculty, fellows, and advanced practice providers (APPs, neonatal nurse practitioners and physician assistants) after development of a comprehensive simulation curriculum. Additionally, a curriculum developed by one of the LISA team fellows to train faculty and fellows to “coach” their colleagues while using the VL for ET intubation or LISA was found to be very helpful. VL quickly became the preferred LISA method. As success was demonstrated, we were able to obtain funding to procure a second VL. Our neonatology fellows were assigned the job of cleaning and making sure the device was kept charged and ready for use. Based on the data collected on “complications,” there was not a single episode of the device not being available for LISA.

Change #2—Reinforce Need for Sucrose Sedation and Swaddling; September 2023
Rationale

As part of the original implementation of LISA in our unit, the choice was made to optimize non-pharmacologic methods of sedation of sucrose solution and swaddling [3] to mitigate the adverse effects of laryngoscopy [13,14,15,16,17,18]. Anecdotally, after LISA was first initiated, an increased need for multiple laryngoscopy attempts was noted for infants greater than 24 h of age and/or greater than 32 w GA [3]. Further investigation revealed inconsistent use of non-pharmacological measures for this procedure.

Implementation

The LISA form was updated so that part of the “time-out” included reminders requiring two providers to “initial” the swaddle and apply sucrose solution for each LISA. The data were tracked. Swaddling methods were altered to ensure good access to chest rise and the ability to auscultate while sufficiently containing the baby for the procedure.

The fellows told us that sometimes swaddling and sucrose application were in fact being done, but it was not being documented. We ultimately had to put two of our fellows on “LISA probation” (they lost their eligibility to perform the procedure for some time) due to documentation/non-compliance for swaddling and sucrose, after which compliance increased remarkably.

Change #3—Addition of Downes Scoring Criteria for LISA; December 2023; modified March 2024
Rationale

We noted that our “LISA failure” rate (intubation within 7 days following LISA), particularly in the first 72 h, was only 14% whereas most of the published literature [19,20] reported post-LISA intubation (“LISA failure”) rates of approximately 30–45%, suggesting that surfactant was being given to infants with mild RDS who could recover without the need for surfactant. On review of the data, many infants, especially those >32 w GA, were receiving LISA while they were on minimal CPAP support (+5 or lower) and <25% FiO_2_ requirement. To try to avoid surfactant administration to babies with no or very mild RDS who might never have needed surfactant, the choice was made to quantify the degree of clinical RDS by implementing Downes scoring [21].

Implementation

**Figure 3:**
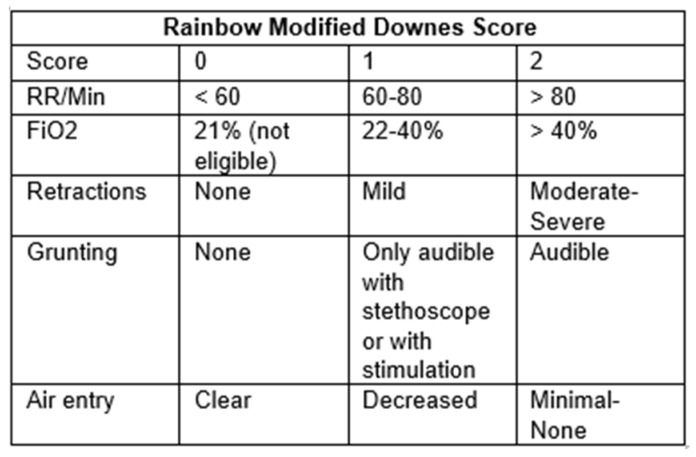
The Rainbow NICU modified Downes scoring rubric that includes a “1” score for audible grunting only when stimulated or with a stethoscope and more specific oxygen requirements than the original [21].

A chart quantifying the Downes scores was used for infants who were candidates for LISA. The infant would be assigned an hourly Downes score by respiratory therapy, bedside nursing, or the medical team; published studies show high inter-rater reliability with this score [22]. Based on the RDS-NeXT recommendations [6], a score of 1–3 was considered for “watchful waiting”; for a score of 4–7, LISA was recommended, implying one should not wait until the score is >7. In our usage, it is suggested that if the score is >7, intubation should be considered. Initially, there was inconsistency about how to score “grunting” when it was only present with stimulation or agitation, versus present at rest. The decision was made to modify the original Downes score for use in our unit to clarify this point, assigning a “1” for when the grunting could only be heard using a stethoscope in the original score, but also for babies who had grunting only when disturbed. We also used more specific oxygen requirements than the simple finding of cyanosis in the original Downes score. Included is the current version of our “Rainbow Modified Downes Score” rubric being used in our unit (Figure 3).

Change #4a—Lowering LISA gestational age limit from 27 weeks to 25 weeks; October 2024
Rationale

When LISA was first started in our unit, the lowest gestation included was 28 w and then was lowered to 27 w. As more data were available in the literature and our LISA proficiency improved, we decreased the lower limit to 25 w. We were reassured to know that the leaders of the OPTIMIST randomized trial, despite the initial caution noted in their paper [23], also had decreased their lower limit to include 25 w (personal communication, RMR with Peter D’Argaville, 2023).

Implementation

After sharing this new information with all NICU medical, nursing and respiratory therapy providers, and updating forms to reflect the lower gestational age, the eligible age of LISA for infants not requiring intubation in the delivery room was lowered from 27 w to 25 w.

Issues with implementation included some disagreement on the timing of LISA due to the potential effect on “Golden Hour” protocols that focus on safety and efficiency during the first hour after birth in babies <28 w. It was decided that surfactant would be prioritized over umbilical line placement, when possible, due to increased efficacy of surfactant with earlier delivery [24,25,26,27]. Additionally, after the first patient of lower gestational age received surfactant unilaterally, a change was also made to reflect the smaller size of these patients, placing the vocal cord guide mark on the catheter at 1.5 cm for infants <28 w GA vs. 2 cm for those ≥28 w. This was the original plan when LISA was started, but since the original lowest gestational age at the start of LISA was 28 w, we used only a 2 cm vocal cord guide mark and this was a serious oversight when we decreased to 25 w to not have added this reminder in the LISA kit instructions.

Change #4b—More aggressive timing of surfactant treatment in general; December 2024
Rationale

**Figure 4:**
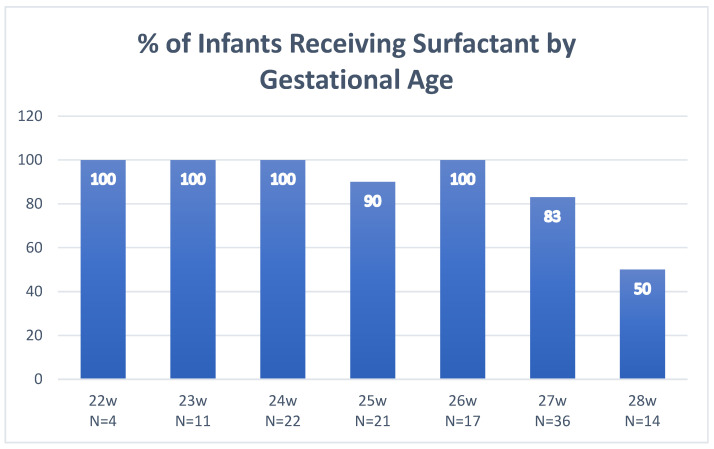
Surfactant usage in previous years. To help determine the best approach of mandatory surfactant in our own NICU, our own patients’ historical requirements at each gestational age were examined. For the infants less than 29 w gestational age born in our unit in 2022–2023, 83–100% of infants <28 w required surfactant with usage dropping to 50% at 28 weeks; therefore, it was decided to make the cutoff for mandatory surfactant at <28 weeks in an effort to ensure earlier surfactant administration for these infants.

**Figure 5:**
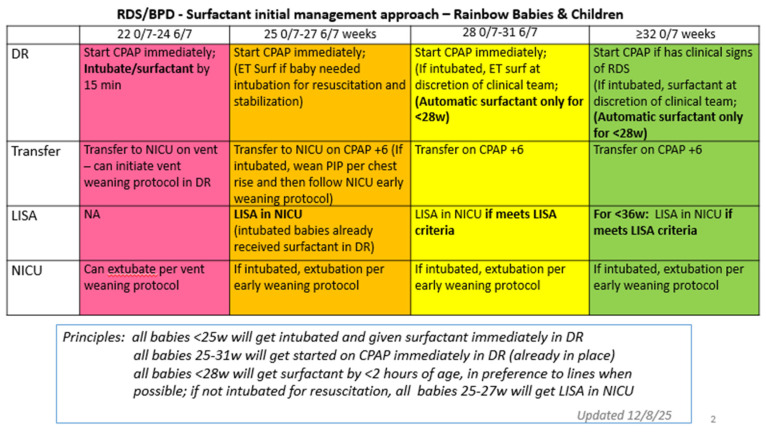
Management of surfactant administration by gestational age. This tool was implemented as part of a push for earlier surfactant delivery in qualifying neonates.

At the same time the GA limit was lowered, it was decided to reconsider surfactant treatment for all Extremely Low Gestational Age Neonates (ELGANs). Literature showed that neonatal intensivists at other NICUs, in the interest of earlier surfactant administration, had a prior planned ETT intubation immediately after birth [28,29] for the lowest GA babies. In addition, anecdotally, significant BPD in our patients at 24–28 weeks who received delayed initial surfactant (after 24 h) was noted. Data were examined regarding the historical need for surfactant in our patients in our NICU (Figure 4) and it was noted that in 83–100%, at all gestational ages <28 w, surfactant was ultimately given. Since babies <27 w were not LISA-eligible at that time, these babies were all being intubated. Therefore, a strategy was agreed upon that would provide surfactant earlier for those babies who ultimately would be getting it anyway. Now, all babies <25 w are intubated in the delivery room and given surfactant via ETT; LISA is mandated after arrival in the NICU for those babies 25–27 w if they did not require intubation for resuscitation (Figure 5).

Implementation

After sharing this information with all NICU medical, nursing and respiratory therapy providers, the change was made to automatic intubation and automatic LISA (Figure 5). This change was remarkably popular immediately. The change to automatically intubate and give surfactant in babies at 22–24 w was met with positivity because it was very clear what the plan would be for these tiny babies to receive surfactant in the delivery room. LISA was prioritized over umbilical line placement for the “Golden hour” stabilization in non-intubated babies <28 w.

## 3. Results

LISA was attempted in 399 babies with surfactant successfully delivered via LISA in 97% (388 babies) between 2020 and 2025, with a mean GA of 31.7 (2.7) and a mean birth weight of 1752 g (590 g) (Table 1). Not surprisingly we noted a significant association with a higher need for intubation despite LISA in the lower GA cohorts. The lower GA infants also had fewer LISA attempts to achieve a successful procedure.

**Figure 6:**
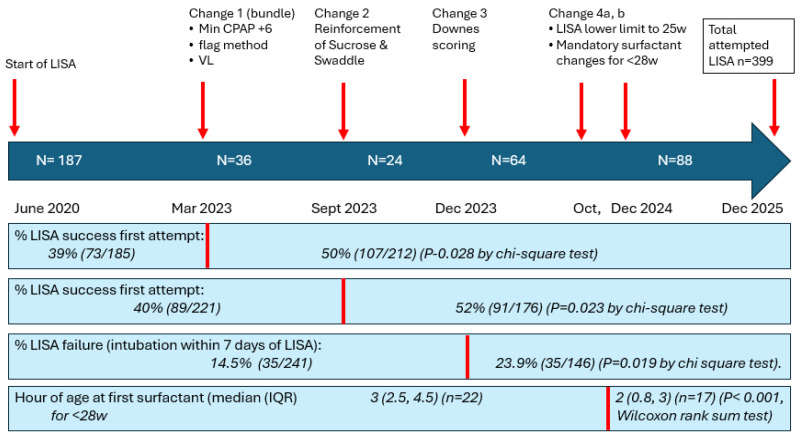
Report of the seven changes made to LISA practice in our NICU since initiation in June 2020 and targeted measurements in all babies before and after each change. Change 1 was a bundle of three changes (using a minimum CPAP of +6 for LISA eligibility, using a “flag,” and encouraging VL for LISA) and was associated with improved first-attempt LISA success from 39% to 50% (*p* = 0.028 by chi-square test). Change 2 reinforced the use of sucrose and swaddling and was associated with improvement in LISA first-attempt success from 40% to 52% (*p* = 0.023 by chi-square test). Introducing Downes scoring to LISA eligibility (Change 3) was associated with a desired increase in “LISA failure” (defined as intubation within 7 days of LISA) confirming a likely prior over-treatment with surfactant. Change 4 was a bundle of 2 changes decreasing the lower limit of LISA to 25 w and introducing a new protocol mandating surfactant by ETT or LISA for babies <28 w. This was associated with earlier surfactant administration in infants <28 w with a decrease in the hours of life surfactant was received from 3 h to 2 h (*p* < 0.001 by Wilcoxon rank sum test). LISA, less-invasive surfactant administration; NICU, neonatal intensive care unit; CPAP, continuous positive airway pressure; VL, videolaryngoscopy; ETT, endotracheal tube; w, weeks.

For each change we examined one specific outcome associated with that change (Figure 6). For change 1a–c, data showed an increase in a successful LISA procedure with only one attempt needed increased from 39% prior to the change to 50% after the change (*p* = 0.028 by chi-square test). For change 2, in examining process rather than an outcome, the documented use of sucrose (from our LISA kit forms) increased significantly from 57% to 94% and swaddling documentation from 62% to 85% (both *p* < 0.0001 by chi-square) after these practices were reinforced. Data showed an increase in a successful LISA procedure with only one attempt needed increased from 40% prior to the change to 52% after this change (*p* = 0.023 by chi-square test). For change 3, Downes scoring was implemented in December 2023 and the modification for intermittent grunting or grunting only audible with a stethoscope was added three months later in March 2024. After implementation of Downes scoring, there was an expected and logical increase in the number of infants requiring intubation within 7 days of LISA (LISA “failures”) by 9.4% from 14.5% to 23.9% (*p* = 0.019 by chi-square test), suggesting that surfactant was likely being administered to some infants who would have recovered without surfactant prior to this change. For change 4a–b, the hope was that instituting a clear plan for each gestational age infant’s surfactant dosing would result in earlier treatment in the ELGANs infant <28 weeks. In babies born <28 w a significant decrease was noted in the hour of life at which the first dose of surfactant was received in LISA infants from a median (IQR) of 3.0 (2.5, 4.5) to 2.0 (0.8, 3), *p* < 0.001 by Wilcoxon rank sum test. The ultimate goal is to have these infants receive surfactant immediately on arrival to the unit and before umbilical line placement, so it is anticipated that this number will continue to improve as this becomes standard practice in the unit.

We further examined the adverse events (Table 1) over time and over gestational age and provide this descriptive information in a supplemental figure. Figure S1a reports the percent of infants with any adverse event/complication by year of LISA. (“20” is the year 2020, and so on). And in Figure S1b we report the percent of infants with any complication by gestational age

**Figure 7:**
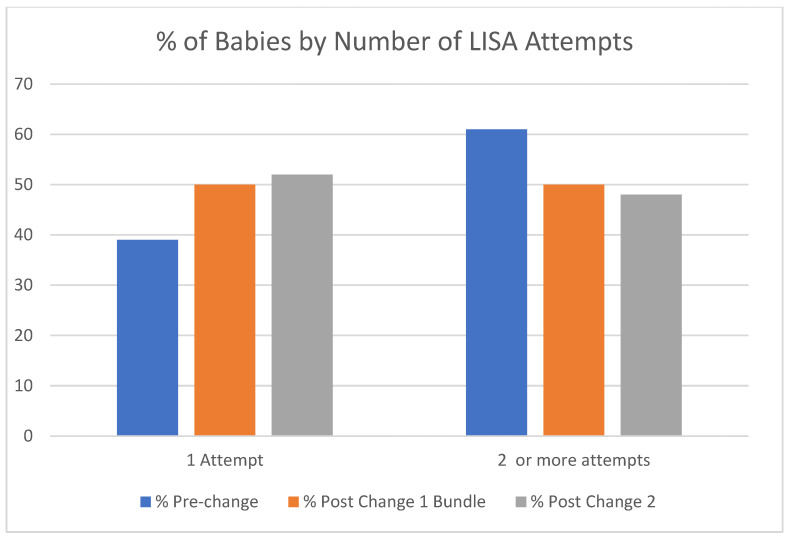
Prior to any changes, LISA’s success on first attempt was 39%. This increased to 50% after the change 1 bundle (increase to CPAP +6 minimum, flag method, introduction of video laryngoscopy) (*p* = 0.028, by chi-square) and increased further to 52% after reinforcement of sucrose sedation/swaddling (*p* = 0.023, by chi-square).

Success with the first attempt at LISA (each time the laryngoscope is inserted is counted as an “attempt”) was measured before and after both the change 1 bundle (included promoting VL) and change 2 (reinforcing sucrose and swaddling). Success increased significantly (*p* = 0.028) by 11% after the change 1 bundle, and by another 2% after the reinforcement of sucrose sedation/swaddling (*p* = 0.023) (Figure 7).

## 4. Discussion

Over the years in most NICUs the goal has been to achieve favorable outcomes for premature babies by minimizing the incidence of BPD. The aim has been to achieve this by decreasing barotrauma, minimizing the time spent on the ventilator, and yet at the same time giving surfactant in a timely manner, preferably within the first 2 h.

For a while, the practice was to place premature babies on CPAP, which sometimes delayed the administration of surfactant as the only method was via ET intubation. In most NICUs, including ours, intubation was performed after sedation medications were given to the baby. This practice led to babies remaining on the ventilator longer than initially desired. LISA seemed to perfectly fill this gap by enabling providers to administer surfactant without the need for ET intubation. However, this practice seemed to be more prevalent in countries other than the USA. As mentioned earlier, we quickly discovered that in 2020, few centers in the USA had well-described protocols for the administration of LISA. Now, anecdotally, over half of third-year fellows reported using LISA routinely in their NICU (show of hands, January 2026, personal communication RMR).

In our previous quality improvement project [12], we were able to show improvement in first attempt intubation using VL especially in less experienced providers. Because the videoscope screen allows several clinicians to simultaneously visually establish appropriate catheter placement into the trachea via the viewing screen, providers are no longer reliant on a single provider confirming tracheal catheter placement via direct visualization. Rather, consensus is gained among several bedside clinicians regarding catheter placement using the viewing screen. This successful teamwork was the basis of our impetus to launch a similar systematic project for surfactant administration via LISA. A key component to the success of this change can be attributed to the establishment of a rigorous VL training for faculty, fellows and the advanced practice provider team. Particularly the workshop at which providers learned to “coach” their peers strengthened both technical and troubleshooting skills. This was a major change in how most of our providers performed intubation. As expected, some “early adopters” embraced the new technology readily while others took more time to become proficient. In any case, we were able to demonstrate better success for trainees/beginners in first attempt intubations, both for our neonatology fellows and particularly our APPs. Excitement, willingness to try VL and ultimately, proficiency increased for many of our LISA and ETT intubators. One of the “selling points” was the availability of the smallest 000 blade of the VL for our tiniest babies and this led some to become “early adopters.” Cost is a concern for some hospitals, but having the data to show greater intubation success [12] helped us to both be able to keep the equipment maintained and repaired as needed, and to procure a second device. Our fellows were given the vital responsibility of making sure the VL was kept cleaned, charged, and properly stored after each use, and this worked well. In tracking lack of VL availability as a “complication” there were zero instances in 399 LISA attempts for which the VL was unavailable.

Over time, it was noted that, uniformly across all gestational ages, the first attempt success rate for LISA in our unit was much lower than our first attempt intubation rate (40% vs. 57%) [12]. This could be attributable to the fact that intubation was done after rapid sequence sedation, which has been shown to improve first attempt success rates and reduce adverse events [13,30,31], whereas LISA was done with the baby awake and breathing. There is no consensus on sedation prior to performing LISA [19,20,32,33,34]. In a randomized trial of Surfactant Administration via Laryngeal mask or Supraglottic Airway (SALSA) vs. ET surfactant, the study was criticized for the ETT arm having medication and the laryngeal mask airway (LMA) arm not [35]. The ET arm had a significantly higher rate of reintubation, likely due to sedation medication. But one of the goals of LISA, or any non-invasive surfactant delivery, is to avoid ETT placement and even brief MV. Therefore, drugs potentially leading to respiratory depression possibly requiring ETT placement/MV do not seem to be the best strategy. This issue is still unresolved but most LISA providers are not using routine sedation other than sucrose [32].

The addition of Downes scoring was added to help ensure we were not overtreating mild cases of RDS. While LISA is a less invasive method of surfactant administration, it does still involve laryngoscopy and its associated risks [8,9,10,11,21,36]. Furthermore, cost effectiveness should always be a consideration. While surfactant administration has been shown to reduce healthcare costs in comparison to CPAP alone [37], and especially in those <32 w GA [38], if it is given to patients who would not progress to have CPAP failure or who have persistently low oxygen requirements, this economic benefit may be diminished. Trying to find the balance between aggressive delivery of surfactant by LISA to the right populations and using LISA too aggressively, especially in moderate or late preterm infants who might have no or mild RDS, has been challenging. Downes scoring was implemented to determine appropriateness for surfactant. The Downes score was first published in 1970 and assigns severity of respiratory distress based on respiratory rate, retractions, cyanosis/oxygen requirements, air entry, and presence of grunting [21]. It was found to correlate nicely with RDS severity. The 2023 RDS-NExT Consensus statement recommended using the Downes score to assist in choosing when to use surfactant treatment by helping to quantify the level of respiratory distress associated with RDS [6]. That team recommended using a Downes score of 4–7 to be one of the criteria to give surfactant (to encourage more surfactant in babies with significant clinical symptoms); if the Downes score is >7, our guideline suggests the provider consider intubation rather than LISA. Fortunately, the natural history of RDS is for clinical severity to increase over the first day, so with repeated Downes scoring we rarely get to a score above 7. Our current practice is to do our first Downes score approximately 30 min after all “hands off” (lines, settling in, etc.) and then hourly as needed. Our goal was to make our Downes scoring as objective as possible, and we acknowledge that we did not validate the modified “Rainbow Downes Score,” but it did help with the consistency of scoring.

The lower limit of GA to provide LISA was a difficult decision for our team. In the literature, LISA is associated with a lower likelihood of death or BPD when compared with MV or CPAP alone [4,5] in infants with a mean gestational age of 28 w, higher survival without severe complications in infants 23–27 weeks [20], increased survivability without worse neurodevelopmental outcomes in 23–26 w infants [39], reduced need for and duration of mechanical ventilation in infants 25–27 w GA [40], and lower risk of pneumothorax, surgery for retinopathy of prematurity (ROP), and intracranial hypertension (ICH) in patients 22–23 w gestation [41]. Additionally, the Optimist-A trial found that while minimally invasive surfactant did not significantly reduce the risk of BPD or death in 25–28 w week infants, it was associated with reduced risk for need for intubation at <72 h of age and fewer days overall on all forms of mechanical respiratory support [23]. A follow-up study showed lower rates of adverse respiratory outcomes in the first two years of life in the infants who received minimally invasive surfactant [42]. However, in this same study there was a small signal for increased mortality in the very earliest gestation infants prompting the authors to include a cautionary statement in their paper which influenced us to hold off initially on LISA for babies <27 w. The concern for increased mortality in the smallest, most immature infants treated with CPAP only was also a concern in the COIN trial [43] but was not for the SUPPORT trial [44]. We eventually felt more comfortable after much more experience and lowered our LISA lower GA limit to 25 w in 2025.

We acknowledge that our cohort spans a wide gestational age range encompassing infants with markedly different clinical risks and trajectories, but, in fact, this is the wide range for which LISA can be used. Aggregating outcomes across this heterogeneity risks masking important subgroup differences but we reported the demographic data for the entire cohort for simplicity. The repeated modifications to inclusion criteria (e.g., lowering the gestational age threshold, mandating surfactant for infants <28 weeks) created systematically different pre- and post-change populations, which could limit the validity of before-and-after comparisons to some degree. However, when the outcome was more likely to be heavily associated with babies <28 w for example, we only looked at that cohort and this was explicitly stated.

**Figure 8:**
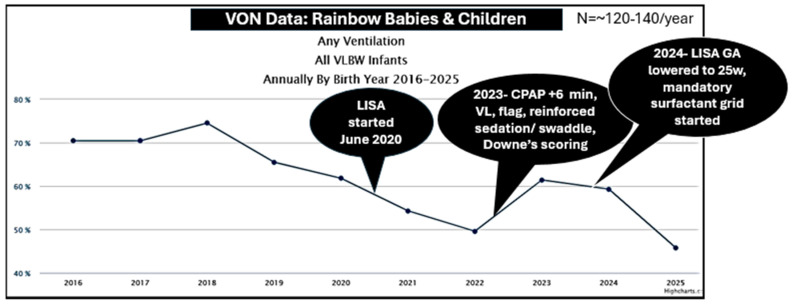
Intubation rates over time. Our Vermont Oxford data (all babies <1500 g or <32 w at birth) show a continued decrease in receiving any intubation/mechanical ventilation for any time during hospitalization for the years 2016–2025.

Although our original intention has been to decrease BPD, we currently have too few highest-risk babies to test this question. Also, our data cohort only includes babies who received LISA. To examine a population-based outcome over time, we report our Vermont Oxford Network (VON) data for “any ventilation” during hospitalization at any time. However, these data are for all VON babies, i.e., <32 w or <1500 g. Our intubation rates were decreasing already but our LISA program does not seem to have increased mechanical ventilation rates (Figure 8). For interest, we added the timeline of the changes described in this paper.

It was very clear to us that we needed to have strong commitment from all the disciplines in the NICU to make this successful. This project has brought our physician and RT teams closer and driven us to a greater sense of shared responsibility to make LISA work well. Also, we realized that we had to make one change at a time and thoughtfully evaluate before proceeding to the next change. However, this was not always possible to do in a strictly real-time timeline due to manpower availability for data collection. Thus, although we considered our changes conceptually in the traditional “PDSA” paradigm, and while we did review our data often, we were not always able to collect all data prospectively to analyze prior to the next change, so we cannot claim changes were directly related to an outcome. It is also possible that a change gets more often adopted over time so its positive effect continues to improve the outcomes (for example use of VL). While we realize our analytical approach in this project did not strictly follow one of the preferred QI models, we felt the data was still important to report for others to understand and use in their own LISA approaches. One of our biggest challenges continues to be thorough documentation on our LISA procedure sheet and requires vigilant follow-up and reminders from the fellows who are generally responsible for collecting the forms and recording the data. We continually evaluate our forms to decrease documentation burden as we continue to streamline our process. Finally, while we were limited to data from a single center, we hope that our transparent reporting of our experience is valuable to another center starting their LISA practice.

## 5. Conclusions

Our unit has seen fewer attempts for a successful LISA, reduced over-treatment with surfactant, and earlier surfactant administration overall in babies <28 w after strategic changes to our established practice were made. We hope this information can be of use to providers in other NICUs as they either implement LISA or continue to fine-tune their practice.

## Figures and Tables

**Table 1:** Demographics of all babies with LISA attempts.

Gestational age (weeks)	31.7 ± 2.7
Birth weight (grams)	1752 ± 590
**LISA procedure**	
LISA attempted	399
LISA procedure successful	388 (97%)
Number of attempts ^ per LISA	
25–27 w 6 d (n = 40) ^a^	1 (1, 2)
28–36 w 6 d (n = 357)	2 (1, 3)
Age (hours) at first LISA (median (QR))	4 (2.5, 9.9)
Pre-LISA FiO_2_	32 (28, 40)
Post-LISA FiO_2_	23 (21, 30)
Adverse events during LISA procedure (n (%))	
Bradycardia	39 (8%)
Desaturation	80 (20%)
Cough	11 (3%)
Surfactant reflux	46 (12%)
Emesis	2 (<1%)
Trauma	17 (4%)
Emergency intubation	8 (2%)
Any adverse event	165 (41%)
GA of all LISA-attempted babies	
25–27 w	40 (10%)
28–32 w	171 (43%)
33–36 w	188 (47%)
Intubation required within 7 days of LISA procedure by GA *	
25–27 w	54%
28–32 w	18%
33–36 w	11%
Total	18%
BPD outcome (NRN 2019 criteria) N = 207 ^b^	
None	61%
Grade 1	20%
Grade 2	15%
Grade 3	2%
Death before 36 w	2%

Data are presented by mean (SD) or median (IQR) or n (%); GA, gestational age, w weeks, SD, standard deviation, IQR, interquartile range; ^ an attempt is counted each time a laryngoscope is placed into the mouth. ^a^ *p* = 0.06 (trend) compared with higher GA, by Wilcoxon rank sum test; ^b^ only determined for babies <32 w or <1500 g; * *p* < 0.0001 by chi-square test.

## Data Availability

Data are not publicly available currently due to patient confidentiality but are available from the authors with all reasonable requests.

## Supplementary Materials

The following supporting information can be downloaded at: https://www.mdpi.com/article/10.3390/children13040571/s1, Figure S1a: Percent of infants with any adverse event/complication by year of LISA for the years 2020–2025. Figure S1b: Percent of infants with any adverse event/complication during LISA, by gestational age, for the years 2020–2025.

## Abbreviations

The following abbreviations are used in this manuscript:

MIST	Minimally Invasive Surfactant Therapy
LISA	Less-Invasive Surfactant Administration
NICU	Neonatal Intensive Care Unit
W	Weeks
GA	Gestational age
CPAP	Continuous positive airway pressure
RDS	Respiratory Distress Syndrome
IQR	Interquartile Range
ETT	Endotracheal tube
RT	Respiratory therapist
QI	Quality improvement
BPD	Bronchopulmonary dysplasia
PDSA	Plan-Do-Study-Act
EtCO_2_	End-tidal CO_2_
VL	Video laryngoscopy
DL	Direct laryngoscopy
MV	Mechanical ventilation
ELGAN	Extremely low gestational age neonate
