# Supplementary material for: Improving LISA Practice: An Ongoing Observational Quality Improvement Initiative Following Initiation of Less-Invasive Surfactant Administration in a Level IV NICU
Source: Children (Basel). 2026 Apr 20;13(4):571. doi: 10.3390/children13040571 (PMC13114399; doi:10.3390/children13040571)
Supplement: Supplementary file 1 [file children-13-00571-s001.zip › children-4158156-supplementary.pdf]

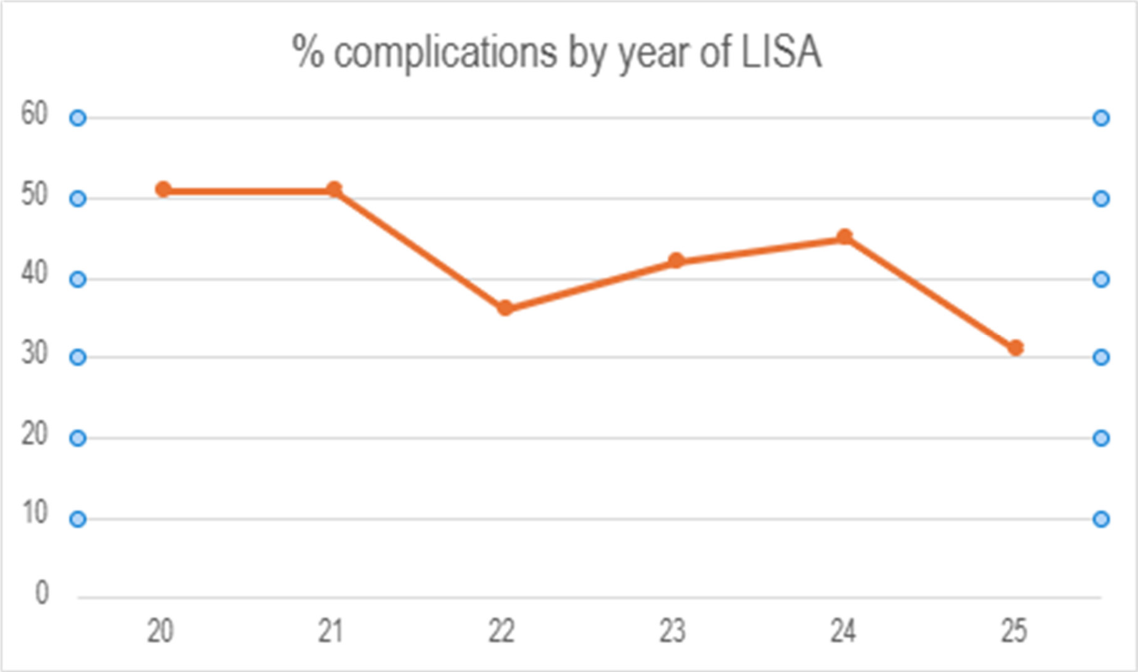

Figure S1. a: Percent of infants with any adverse event/complication by year of LISA for the years 2020-2025.

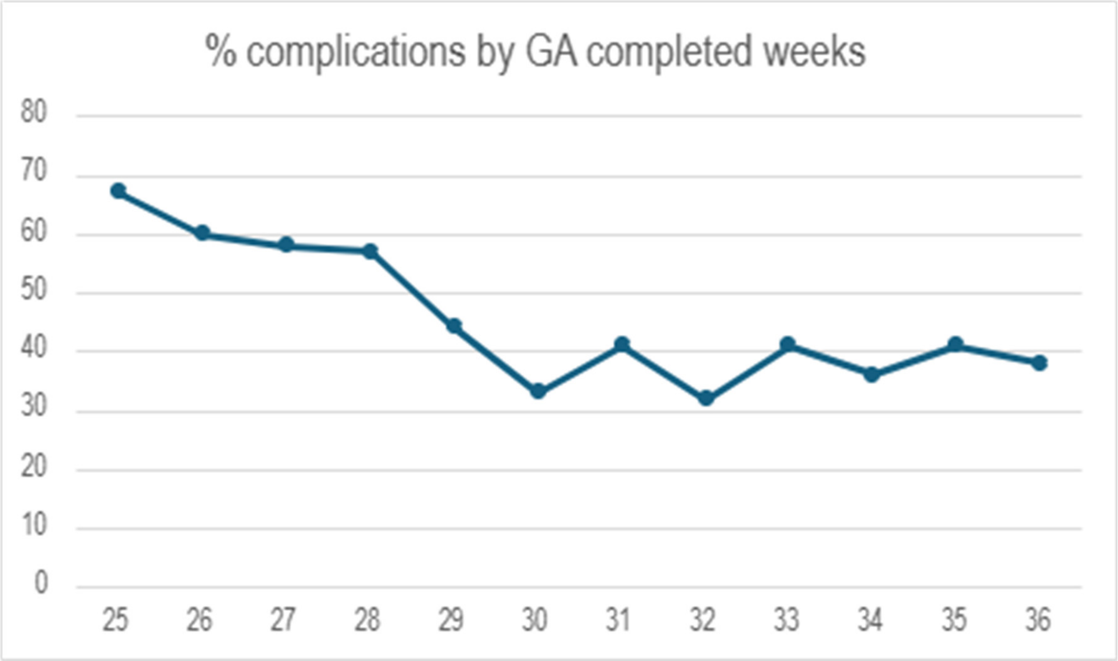

Figure S1b: Percent of infants with any adverse event/complication during LISA, by gestational age, for the years 2020-2025.
